# How Preoperative Closed Reduction and Time to Surgery Impact Postoperative Palmar Inclination in Distal Radius Fractures

**DOI:** 10.3390/jcm13082316

**Published:** 2024-04-17

**Authors:** Frank Beyer, Johannes Oppermann, Tobias Prasse, Lars Peter Müller, Peer Eysel, Jan Bredow

**Affiliations:** 1 Department of Orthopedics and Trauma Surgery, Krankenhaus Porz am Rhein, Urbacher Weg 19, 51149 Cologne, Germany; j.bredow@khporz.de; 2 Department of Orthopedics and Trauma Surgery, Medical Faculty, University Hospital of Cologne, Kerpener Strasse 62, 50937 Cologne, Germany; johannes.oppermann@uk-koeln.de (J.O.); tobias.prasse@uk-koeln.de (T.P.); lars.mueller@uk-koeln.de (L.P.M.); peer.eysel@uk-koeln.de (P.E.)

**Keywords:** radius, fracture, palmar inclination, reposition

## Abstract

**Background:** The anatomical reconstruction of the wrist is the aim when treating distal radius fractures. Current literature on the importance of preoperative reduction in fractures that are treated operatively is limited. **Methods:** This study investigated the effect of the preoperative closed reduction of distal radius fractures on the day of trauma and the time to surgery on postoperative palmar inclination. A total of eighty patients (48 females and 32 males, mean age 55.6 years) were studied retrospectively. All patients were treated with an open reduction and internal fixation. The palmar inclination angle was measured using X-rays by two investigators, and the interobservers and pre- and post-reduction parameters were compared. **Results:** When the surgical management of closed distal radius fractures is required, neither initial repositioning nor a delay of up to 14 days to the surgical treatment influences postoperative palmar inclination. **Conclusions:** The significance of preoperative reduction of distal radius fractures without neurovascular or extensive soft tissue damage is limited and is not leading to improved outcomes. When surgery is about to be performed, surgeons should carefully consider if reduction is really vital preoperatively. Level of evidence: III.

## 1. Introduction

Distal radius fractures are the most common type of upper extremity fractures, making the analysis of different treatment techniques clinically important [1]. Typically, occurring after falling on the hand or wrist, distal radius fractures account for a significant proportion of bone injuries, particularly in the elderly and individuals with osteoporosis [2].

Radius fractures affect all age groups. In young people (below the age of 40 years), they are most often related to high-energy traumas (falls or motor vehicle accidents) [3,4]. Within this age group, males are 40% more often affected than in the corresponding female age group [5]. In these cases, two-thirds of the fractures include the articular surfaces, and more than half of them are known to be significantly displaced [6].

Multiple classifications exist for distal radius fractures. Historically described fractures include the following: Colles fracture (extension fracture with dorsal dislocation), Smith fracture (flexion fracture with volar dislocation), Barton fracture (intraarticular two-part fracture), Reversed Barton fracture (also Smith type II, intraarticular with volar fragment), and Hutchinson or Lorrie fracture (intraarticular fracture with affection of the styloid process of the radius) [4,7,8,9]. Nowadays, the classification of the Association of Osteosynthesis (AO) is more common. In terms of articular fragments, the following five fragments are most typical: volar ulnar corner, dorsal ulnar corner, dorsal wall, the free-intraarticular fragment, and the radial styloid fragment [10]. Based on a combination of that knowledge and thorough radiographic analysis of the given fracture morphology, certain strategies are used to reduce radial fractures intraoperatively [10].

When classifying distal radius fractures, numerous factors are described in the literature to be criteria for instability. In general, age is listed not only as a risk factor for distal radius fractures, but is also known to be a strong factor when predicting recurrent fracture dislocation and complications such as malunion [11]. Additionally, fractures that can only be immobilized in an extreme position are considered to be unstable [12]. Moreover, radial shortening, a difference of seven or more degrees in the frontal plane, a radial inclination of less than ten degrees in anteroposterior projection radiographically, and a relative lengthening of the ulna greater than four millimeters are thought to be indicators for instability [11,13]. Further important criteria are the discontinuity of the palmar joint part, fragments of the dorsal or volar edge of the radius, comminuted fractures with a relevant shortening of the radius, a dissociation of the radius and ulna, and the tendency for a recurrent dislocation after reduction [11]. On lateral radiographs, a tilt of a fragment greater than 20 degrees dorsally and also a tilt of a fragment of more than 20 degrees in the opposite direction (ventrally), is considered to represent an unstable fracture [13,14].

Many fractures can be treated conservatively using casts and the temporary immobilization of the affected arm. However, cases of more severe injuries require surgical treatment. Indications for the operative treatment of distal radius fractures are closely related to the abovementioned instability criteria. In addition to unstable fractures and dislocated intraarticular fractures, fractures that are accompanied by higher degrees of soft tissue damage, as well as open fractures, are typically managed through surgical intervention. Furthermore, the traumatic compression of the median nerve, repeatedly unsuccessful reduction, acute ischemia or a disturbance of the blood supply, and complex carpus or wrist injuries make an operative treatment necessary. Relative indications for operative treatment are not supported by evidence-based guidelines, but according to expert consent. Patients who demand a highly functioning wrist for personal or professional reasons may require surgery even if the listed criteria do not apply. Additionally, parallel injuries of the lower extremities or synchronous injuries, or multiple injuries requiring local operative treatment close to the distal radius fracture site, are several of the relative indications for surgical treatment. 

Operative strategies required in the aforementioned cases include external fixation and a variety of internal fixation techniques, and are often required in the abovementioned cases [15]. For radius fractures that include the articular surface, operative approaches involving open reduction and internal fixation (ORIF) have become increasingly common [15,16]. Prior to the surgical treatment, one should thoroughly assess the patient’s history and the exact trauma mechanism, since this can already provide valuable information on the fracture morphology and consequently on the individual treatment path. In cases of intraarticular distal radius fractures, computer tomography can also be beneficial to assess the fracture more in detail and properly plan the surgery.

The preoperative reduction of radial fractures that require operative treatment can be performed to restore the position of the bone to its anatomical alignment, or as close to it as possible. If successful, fracture reduction has multiple advantages. The most important upside is to minimize the mechanical stress and strain that is caused by a dislocated fracture as it leads to swelling, edema, and in turn can impair the surrounding structures like nerves and soft tissue [17]. Preoperative reduction is also thought to optimize the intraoperative conditions, since less reduction is necessary intraoperatively, and the best radial position can be achieved, leading to an ideal fracture healing and reduced risk of an undesirable non-anatomical position. The reduction is not needed for aesthetic reasons, but rather because proper alignment is known to be crucial in terms of the functionality of the wrist [17]. Proper alignment should be achieved to prevent long-term complications like wrist arthrosis, decreased joint mobility, and chronic pain. If performed, sufficient analgesia is crucial. The injection of local anesthetics at the fracture site can be performed safely and effectively [18,19]. In addition, no evidence exists on which intraoperative anesthetic modality is most preferable during the surgical treatment of distal radius fractures [20]. Post-reduction radiographs are mandatory for the verification and documentation of a proper reduction of the fracture.

However, the intraoperative reduction makes the surgery easier, since it allows a better approach to the fracture and reduces complications related to intraoperative soft tissue damage.

Nevertheless, the proper treatment choice remains controversial, and clear guidelines do not exist yet [21,22]. Bone density and the activity level of the individual should always be considered, especially in the elderly patients [23]. Also, a second attempt of fracture reduction in radius fractures with dorsal comminution results in worsened comminution, even if the general alignment of the fracture improves [24].

In daily clinical practice, patients with a closed distal radius fracture without neurological symptoms are normally treated after the regression of the soft tissue swelling within 14 days after injury. The effect of preoperative repositioning on the day of trauma is unclear, and waiving the reduction does not result in worse outcomes [25].

This study investigated the influence of preoperative repositioning as well as the time between fracture and operation on the postoperative palmar inclination (PI). In addition, this paper aims to discuss the rationale, techniques, and outcomes associated with the preoperative repositioning of distal radius fractures, drawing from a variety of studies and clinical guidelines available in the medical literature.

## 2. Materials and Methods

### 2.1. Experimental Setup

With an institutional review board approval by the medical ethics committee of the University of Cologne (ethics number 15-402), we investigated all patients with AO 23-A2, 23-A3, or 23-C1-3 distal radius fractures who were treated operatively between January 2012 and December 2013 in our department. The study design was retrospective. All patients included in the study had distal radius fractures, and reduction was performed on the day of injury.

The injection of a local anesthetic at the fracture site (Mecain^®^ 10 mg/mL mepivacainehydrochloride, PUREN Pharma GmbH & Co. KG, Munich, Germany) was performed using C-arm fluoroscopy, which has previously proven to be a safe and reliable tool in fracture reduction [26]. The reduction was achieved with the patient being positioned in a horizontal position with a 90-degree shoulder abduction and a 90 degree flexed elbow joint. As used for various purposes in the current literature, the fingers of the injured arm were placed in a traction device (comparable with a Chinese finger trap), thereby trapping the fingers [27]. Gravity causes the pulling of the arm toward the ground, resulting in a tightening of the fingers in the traction device. 

Two groups were studied: an initial reduction group that underwent repositioning on the day of the injury, and a non-reduction group treated with cast immobilization without repositioning until surgery. The outcomes of all the patients that could be included during the period of inclusion were studied based on the presence of gapless documentation, pre- and postoperative X-rays, and a post-reduction X-ray (if available). Preoperative reduction was performed to restore the anatomical alignment as far as possible in cases of clinical nerve or vessel damage, impending skin perforation, or gross deformity.

### 2.2. Patient Characteristics

Eighty patients (48 women and 32 men, respectively) with a mean age of 55.6 ± 17.2 years were retrospectively analyzed. The mean age of men was 48.4 ± 17.8 years. Males were significantly younger than women (60.6 ± 15.0 years) (*p* = 0.002). The outcomes of all 80 patients with gapless documentation, pre- and postoperative X-rays, and, if available, X-rays after closed reduction were studied.

### 2.3. Data Analysis

**Figure 1:**
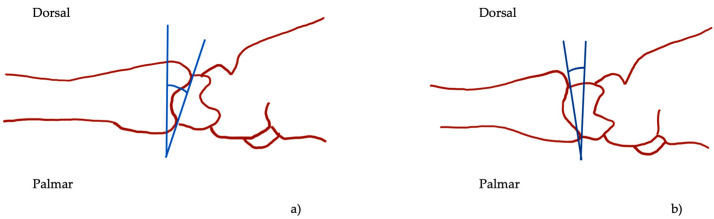
Palmar inclination (blue angle, **a**) and dorsal tilt (blue angle, **b**) in lateral view as illustrated by the angle between the two blue lines.

The PI measurements on X-rays were made using Impax EE (Agfa HealthCare GmbH, Bonn, Germany). The PI was determined as the angle between a line connecting the dorsal and palmar lips of the articular surface of the distal radius and a line perpendicular to the central axis of the radius on the lateral view (Figure 1) [28]. Negative values were defined as dorsal and positive values as palmar tilt. Two investigators (FB and JB) each performed all measurements.

### 2.4. Statistical Methods

The normal distribution of the data was validated, using the Kolmogorov–Smirnov procedure. Radiographic alignment parameters were measured in a random order to avoid bias. The parameters were compared before and after reduction with corresponding parametric and non-parametric tests. The significance level was set at *p* < 0.05.

The mean values of measurements were calculated and used in further calculations. Interobserver reliability was assessed via intraclass correlation coefficient (ICC) in a two-way mixed model with absolute agreement. Values less than 0.40 were considered poor, between 0.40 and 0.59 fair, between 0.60 and 0.74 good, and excellent values were defined between 0.75 and 1.00.

Statistical analysis was performed using SPSS 29 (SPSS Inc., IBM Company Headquarters, Chicago, IL, USA).

## 3. Results

### 3.1. Radiological Measurements

A2 fractures occurred in 7.5% of the cases, A3 fractures in 13.8%, C1 fractures in 3.8%, and C2 fractures in 16.3%, while C3 fractures were most common, occurring in 58.8% of patients. Overall, the preoperative radiocarpal articular surfaces showed an average dorsal tilt of 14.9° ± 18.7°. The postoperative mean palmar inclination was 4.9° ± 6.2°. An average improvement in the sagittal plane of 19.8° ± 16.9° was achieved. 

Preoperative angles differed significantly depending on fracture severity (*p* = 0.024), but the dependence of postoperative PI on initial fracture severity did not achieve significance (*p* = 0.073). 

**Figure 2:**
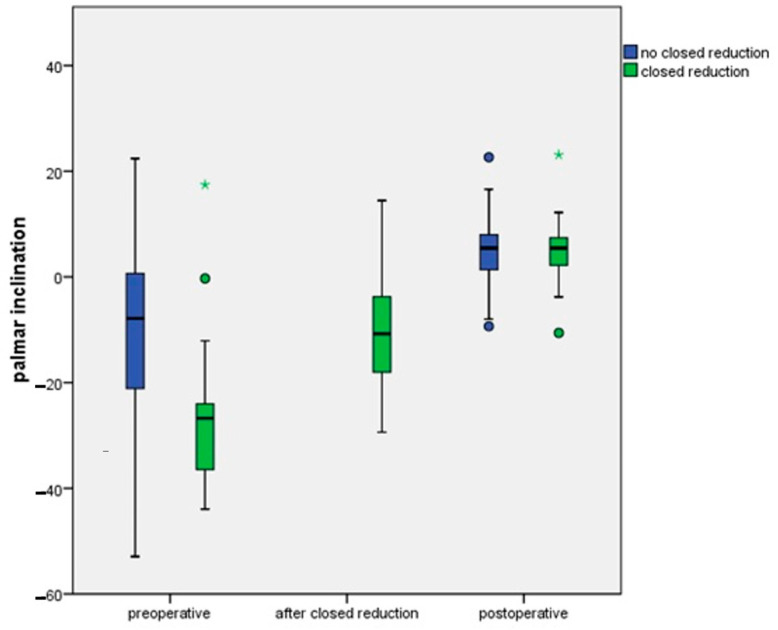
The palmar inclination shown preoperatively, after closed reduction, and postoperatively. Differences between postoperative and preoperative palmar inclination, and between the preoperative situation and after closed reduction at day of trauma, are not significant (*p* > 0.05).

Dorsal tilt in the initial closed reduction group (n = 23) was 26.8° ± 3.2° before repositioning and 10.3 ± 9.8° after repositioning, while in the non-reduction group the mean dorsal tilt was 10.7° ± 2.4°. The difference in preoperative dorsal tilt was not significant between both groups (*p* = 0.747). The postoperative mean palmar inclination was 4.9° ± 0.8° in the non-reduction group and 5.0 ± 1.5° in the initial closed reduction group (*p* = 0.935, Figure 2). The initial PI in both preoperative groups was not significantly different when comparing these groups (*p* > 0.05).

A subgroup analysis of C3 fractures showed significant differences in the dorsal tilt between both groups after trauma (*p* < 0.001). After closed reduction, the mean dorsal tilt was 14.9 ± 7.3°, while in the non-reduction group, the dorsal tilt was −10.7 ± 2.9°; the difference was not significant (*p* = 0.267). The values by all fracture types are shown in Table 1.

Postoperatively, the mean PI in the non-reduction group was 4.2° ± 0.8° and 1.1° ± 1.9° in the initial reduction group (Figure 2).

All listed patients in Table 1 underwent operative treatment. Table 1 lists the cases according to their fracture severity and the related pre- and postoperative inclination, as well as before and after reduction (if performed). The last two columns depict the difference when comparing pre- to post-surgery as well as the difference from the ideal inclination.

### 3.2. Time and Palmar Inclination

**Figure 3:**
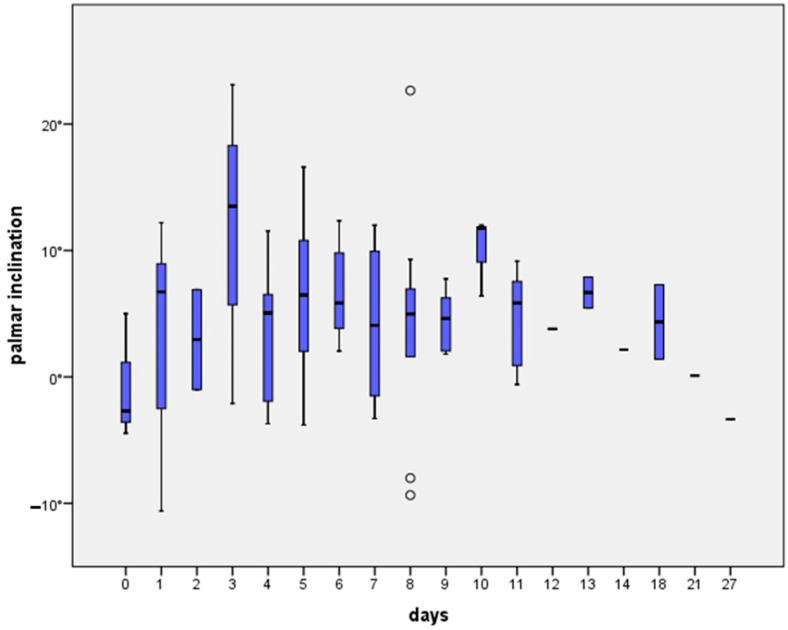
Postoperative palmar inclination depending on interval to surgery. Differences are not significant (all *p* > 0.05).

The postoperative PI showed no significant difference regarding days to surgery (*p* = 0.602). The mean time between trauma and surgery was 7 ± 4.7 days. In the reposition group, the time to surgery was 4.8 days ± 2.9 days, and in the non-reposition group the time to surgery was 7.8 days ± 4.9 days, which was significantly different (*p* = 0.007). Surgical treatment was achieved as soon as possible, but, especially in patients that needed further treatment or consultations before surgery, delays could be observed. At a level-one trauma center, emergency surgeries contributed to the delay of the definitive open reduction and fixation of the radius fractures. Consequently, if delays occurred, they were random due to the described circumstances. The time between accident and surgery did not differ across fracture types (*p* = 0.064, Figure 3). In a subgroup analysis of C3 fractures, no significant influence of delay to surgery on PI was found (*p* = 0.924).

### 3.3. Interobserver Reliability

The preoperative measurements show an ICC of 0.922 with a 95% coincidence interval of 0.988–0.995. For the post-reduction measurements, an ICC of 0.990 (0.977–0.996) was found. The postoperative measurements showed an ICC of 0.971 (0.955–0.981).

## 4. Discussion

Distal radius fractures are among the most common orthopedic injuries encountered in clinical practice. The management of these fractures often involves preoperative repositioning, a step that can significantly influence the outcomes of surgical intervention. These fractures often result from falls on outstretched hands and can vary in complexity from simple, non-displaced fractures to complex, intra-articular fractures. Immediate reduction can alleviate pain and reduce swelling, making the overall management more comfortable for the patient. As a result of this, the proper alignment of the fracture before surgery can lead to better surgical conditions, including a lower risk of intra- and postoperative complications. Restoring the function of the wrist through repositioning is essential, and although less significant, the aesthetic appearance of the forearm is improved as well. There are several preoperative reduction techniques for the reduction of distal radius fractures, including different closed-reduction and traction techniques. The choice of technique often depends on the type and severity of the fracture, and the patient’s health status. The surgeon’s personal choice based on his or her experience is also a factor. Closed reduction is unnecessary for less severe, non-displaced, or minimally displaced fractures [25]. Traction techniques are applied in more complex fractures, especially those with significant displacement. Traction techniques may be necessary to align the fragments properly before surgery. It involves manually manipulating the bone fragments into place without surgical incision.

Besides the presentation of the current literature and guidelines when treating distal radius fractures, our group analyzed the effect of preoperative reduction and the time to surgery on the postoperative outcome. We found that neither preoperative reduction nor the duration of the interval to surgery up to 14 days after injury were observed to have a statistically significant effect on the postoperative PI. In terms of the delay to surgery, our results are in accordance with findings from Howard et al., who analyzed the effect of the delay of surgery on the patient outcome at 12 months after the injury [29]. The authors found that a delay to surgery did not impact the patient’s outcomes negatively. While preoperative repositioning is beneficial, it is not without challenges. Complications can include neurovascular damage, the further displacement of fracture fragments, and rare cases of compartment syndrome. Therefore, the procedure must be performed by skilled practitioners and with appropriate anesthetic support.

To the best of our knowledge, the effect of a preoperative reduction has not been conclusively determined, despite being generally accepted. In clinical practice, the question of whether repositioning on the day of trauma is necessary has yet to be answered. Nevertheless, distal radius fractures with a higher degree of dislocation generally dispose primary physicians to manual reduction. Our postoperative PI results, 95% of which fall within the generally accepted tolerance area [13,30,31,32,33,34], do not take this into consideration. In addition, our groups differ in time to surgery, which makes them harder to compare on the one hand. On the other hand, the mean time to surgical treatment in the reposition group was three days shorter, highlighting that not even a delay to surgery in addition to no reposition results in worse radiographic outcomes.

Clear indications for the preoperative reduction of distal radius fractures are especially those fractures that cause soft tissue, nerve, or vascular complications. Here, reduction is required to prevent secondary damage [35].

Leone et al. examined predictors for repeated dislocation. The degree of radial shortening and a dorsal tilt of over 10° were significantly predictive of early instability one week after surgery. Radial inclination under 10°, radial shortening, age, and palmar tilt were predictors for a late instability (at six weeks) [13]. The AAOS recommendations for an operative approach are radial shortening > 3 mm, dorsal tilt > 10°, or an intra-articular step over 2 mm [35]. Despite these predictors, it should be kept in mind that patients with distal radius fractures are at high risk for developing a complex regional pain syndrome (CRPS) type I. Any unnecessary manipulation during preoperative repositioning should be avoided. Roh et al. found that high-impact injuries, severe fractures, and a female sex are factors that contribute to developing a CRPS after surgical intervention [36]. The explicit influence of manipulation has not been studied.

Controversy exists regarding the best choice of treatment (conservative vs. operative), and, if surgery is preferred, the appropriate time to perform surgery is debated. Even if most of the study results were positive in favor of ORIF [37], a comparative study of ORIF and cast immobilization that showed functional improvement in the early postoperative phase of an operative treatment did not settle matters: after 12 months, no significant difference in wrist function was observed [30]. What is generally accepted in the surgery scenario is that soft-tissue swelling should be minimal and fracture fragments should not have begun to consolidate. What can be stated based on the current literature is that the treatment of distal radius fractures should be adjusted to patients’ individual, functional requirements. An important treatment goal is the recovery of the palmar inclination (PI), while outcomes including a radial shorting of <2 mm, a dorsal tilt of <5°, and an intra-articular step-off or gap < 2 mm are considered acceptable [30,32,37,38,39,40,41,42,43,44,45,46].

The preoperative reduction of distal radius fractures is a critical aspect of orthopedic care that significantly influences patient outcomes and overall treatment success [21]. We want to underline the importance of preoperative reduction in distal radius fractures, considering its impact on functional recovery, complications, surgical ease, and long-term prognosis. 

One of the primary reasons for emphasizing preoperative reduction is the restoration of joint congruity [4,17]. Functional recovery is closely tied to the restoration of normal anatomy, and preoperative reduction plays a pivotal role in achieving this goal. Maintaining the appropriate length, alignment, and angulation of the distal radius is essential for regaining optimal wrist motion and strength [47]. Patients with well-reduced fractures are more likely to achieve a better range of motion and functional outcomes compared to those with suboptimal repositioning [14]. Complications associated with distal radius fractures, such as malunion or nonunion, are significantly reduced when preoperative reduction is meticulously performed [48]. The malalignment of the fracture fragments can lead to joint incongruity, affecting load distribution and contributing to postoperative complications. By ensuring proper repositioning beforehand, surgeons can minimize the risk of long-term complications and enhance overall patient satisfaction [48]. From our experience, surgical ease is another critical aspect influenced by preoperative reduction. A well-reduced fracture provides surgeons with a clearer roadmap during the surgical procedure, making it easier to secure fixation and achieve stable internal fixation. This might not only reduce the length of surgery, but also enhances the precision of the procedure, contributing to a possibly better postoperative outcome. Furthermore, the importance of preoperative reduction extends beyond immediate postoperative results. Long-term prognosis and the development of post-traumatic arthritis are closely linked to the quality of the initial reduction. Anatomical repositioning minimizes joint incongruity, reducing the risk of degenerative changes and preserving joint function over time [4].

The decision-making process regarding the need for preoperative reduction involves various factors, including fracture type, patient characteristics, and surgeon expertise. In cases of displaced or unstable fractures, preoperative reduction is often crucial to achieving the best possible outcome. Non-displaced fractures may not require the same level of preoperative manipulation, but careful consideration is necessary to ensure optimal healing and functional recovery.

In conclusion, the importance of preoperative reduction in distal radius fractures cannot be overstated. It directly impacts functional recovery, reduces complications, enhances surgical ease, and influences long-term prognosis. Surgeons must carefully assess each case, considering the fracture characteristics and patient factors to determine the necessity of preoperative repositioning. Ultimately, meticulous attention to achieving anatomical reduction lays the foundation for successful surgical interventions and improved patient outcomes in distal radius fractures.

One of the limitations of this retrospective study is that we did not compare the postoperative PI with that of the intact side (since this did avoid further patient exposure to radiation). However, 95% of the postoperative PI values fall within the generally accepted tolerance area [13,30,31,32,33,34]. Another limitation is that the comparability could have been affected by the decision of which fractures were reduced. Assumably, rather severe fractures get reduced so as to improve the position, which could have influenced the postoperative angles and pain levels. Our tests could have failed to show an improvement of the reduction group since the comparison was made to a group that did not undergo fracture reduction. Importantly, we did not match our results with patient-reported outcome measures that are an important factor when comparing different techniques. After severe radius injures that require surgical treatment, the patients’ satisfaction, degree of disability, and life quality are crucial in order to evaluate the treatment success. 

Palmar inclination is an accepted parameter to postoperatively assess anatomical restoration of the distal radius. Other studies have shown that radiographic outcomes do not correlate with clinical outcomes [38,49,50], while a prospective cohort study of patients over the age of 50 showed that self-reported outcomes and the “acceptability” of radiographic fracture reduction are unrelated [51].

## 5. Conclusions

Our study shows that the preoperative repositioning of distal radius fractures did not have an impact on postoperative joint integrity and radiographic outcome, highlighting that there is no benefit of preoperative reduction. This does not include reduction in cases of neurovascular or soft-tissue damage that requires immediate treatment. Furthermore, the time to surgical treatment did not influence the radiographic outcome.

## Figures and Tables

**Table 1:** Sagittal inclination of the radiocarpal articular surface. Preoperative, post reduction, and postoperative mean angles with standard deviation according to fracture type classified following the AO classification. Negative values show dorsal tilt and positive values show palmar tilt. The values are depicted in degrees.

	Pre-op	Reduction (n)	Post-op	Pre-Reduction (n)	Post-Reduction	Difference Pre–Post Surgery	Difference from Ideal
A2 (n = 6)	−35.0 ± 8.4	−12.5 ± 8.6 (3)	3.3 ± 2.2	23.7 ± 12.4 (3)	19.0 ± 9.0 (3)	38.3 ± 7.1	6.7 ± 2.2
A3 (n = 11)	−17.9 ± 4.2	−7.8 ± 1.8 (5)	7.1 ± 2.3	17.2 ± 5.0 (5)	16.6 ± 5.2 (5)	25.0 ± 4.2	2.9 ± 2.3
C1 (n = 3)	2.6 ± 6.4	--- (1)	14.1 ± 4.4	--- (1)	--- (1)	11.5 ± 2.0	4.1 ± 4.4
C2 (n = 13)	−7.0 ± 4.1	−2.0 ± 8.4 (3)	6.3 ± 1.7	7.9 ± 5.7 (3)	8.6 ± 5.5 (3)	13.3 ± 3.2	3.6 ± 1.7
C3 (n = 47)	−15.0 ± 2.8	−14.9 ± 2.4 (9)	3.6 ± 0.8	18.4 ± 4.2 (9)	16.0 ± 2.6 (9)	18.6 ± 2.6	6.3 ± 0.8

## Data Availability

All data are available upon reasonable request.
